# Nephron mass determines the excretion rate of urinary extracellular vesicles

**DOI:** 10.1002/jev2.12181

**Published:** 2022-01-22

**Authors:** Charles J. Blijdorp, Thomas A. Hartjes, Kuang‐Yu Wei, Martijn H. van Heugten, Dominique M. Bovée, Ricardo P.J. Budde, Jacqueline van de Wetering, Joost G.J. Hoenderop, Martin E. van Royen, Robert Zietse, David Severs, Ewout J. Hoorn

**Affiliations:** ^1^ Department of Internal Medicine, Division of Nephrology and Transplantation Erasmus MC, University Medical Center Rotterdam Rotterdam The Netherlands; ^2^ Department of Pathology Erasmus Medical Center, University Medical Center Rotterdam Rotterdam The Netherlands; ^3^ Department of Radiology and Nuclear Medicine Erasmus Medical Center, University Medical Center Rotterdam Rotterdam The Netherlands; ^4^ Department of Physiology Radboud University Medical Center Nijmegen The Netherlands

**Keywords:** creatinine, excretion rate, exosomes, kidney, kidney function, nephrectomy, normalization, quantification

## Abstract

Urinary extracellular vesicles (uEVs) are emerging as non‐invasive biomarkers for various kidney diseases, but it is unknown how differences in nephron mass impact uEV excretion. To address this, uEV excretion was measured before and after human kidney donor nephrectomy and rat nephrectomy. In male and female donors, uEVs were quantified in cell‐free spot and 24‐h urine samples using nanoparticle tracking analysis (NTA), EVQuant, and CD9‐time‐resolved fluorescence immunoassay. Female donors had significantly lower total kidney volume (TKV) and excreted 49% fewer uEVs than male donors. uEV excretion correlated positively with estimated glomerular filtration rate (eGFR), creatinine clearance, and TKV (R's between 0.6 and 0.7). uEV excretion rate could also be predicted from spot urines after multiplying spot uEV/creatinine by 24‐h urine creatinine. Donor nephrectomy reduced eGFR by 36% ± 10%, but the excretion of uEVs by only 16% (CD9+ uEVs ‐37%, CD9‐ uEVs no decrease). Donor nephrectomy increased the podocyte marker WT‐1 and the proximal tubule markers NHE3, NaPi‐IIa, and cubilin in uEVs two‐ to four‐fold when correcting for the nephrectomy. In rats, the changes in GFR and kidney weight correlated with the changes in uEV excretion rate (*R* = 0.46 and 0.60, *P *< 0.01). Furthermore, the estimated degree of hypertrophy matched the change in uEV excretion rate (1.4‐ to 1.5‐fold after uninephrectomy and four‐fold after 5/6^th^ nephrectomy). Taken together, our data show that uEV excretion depends on nephron mass, and that nephrectomy reduces uEV excretion less than expected based on nephron loss due to compensatory hypertrophy. The major implication of our findings is that a measure for nephron mass or uEV excretion rate should be included when comparing uEV biomarkers between individuals.

## 1 INTRODUCTION

Urinary extracellular vesicles (uEVs) are nanosized membrane vesicles excreted by cells of the kidney and urinary tract. They are derived either from fusion of multivesicular bodies with the cell membrane (“exosomes”) or from direct outward budding of the cell membrane (microvesicles and apoptotic bodies) (Karpman et al., 2017). Because the uEV proteome and transcriptome contain many disease‐associated proteins and transcripts, uEVs are being explored for non‐invasive biomarkers of kidney function, kidney disease and urological disease (McKiernan et al., 2018; Salih et al., 2014). uEV biomarker discovery has been pursued in patients with acute kidney injury (Panich et al., 2017; Zhou, Pisitkun, et al., 2006), polycystic kidney disease (Hogan et al., 2009; Salih, Demmers, et al., 2016), glomerular disease (Morikawa et al., 2019), and tubulopathies (Dominguez et al., 2017; Salih, Fenton, et al., 2016). In addition, uEVs have been analyzed to identify biomarkers for kidney transplant function and rejection (Braun et al., 2020; El Fekih et al., 2021). Despite the myriad of proteins detectable in uEVs, several methodological questions regarding uEV research must still be addressed prior to clinical application.

It would be preferable to analyze uEV biomarkers in spot urines because of clinical throughput and because it carries a lower risk of EV cargo degradation (Zhou, Yuen, et al., 2006). Therefore, we recently compared several approaches to normalize, quantify, and characterize uEVs directly in spot urines (i.e., without the need for uEV isolation) (Blijdorp et al., 2021). In this previous study, uEVs were quantified and characterized using nanoparticle tracking analysis (NTA), a CD9–based time‐resolved fluorescence immunoassay (CD9–TR‐FIA), and a recently developed method called EVQuant (Hartjes et al., 2020). Using these methods, we identified positive intra‐individual and inter‐individual correlations between spot urinary creatinine and uEV concentration in various settings, including kidney disease (Blijdorp et al., 2021). Accordingly, we proposed that urinary creatinine can be used to normalize uEV proteins in spot urines. A still unresolved question, however, is whether uEV excretion depends on nephron mass and what the effect of nephron loss is on uEV excretion (Erdbrügger et al., 2021). As a large fraction of uEVs are excreted by kidney epithelial cells, nephron loss would be expected to evenly reduce uEV excretion.

Therefore, in the current study, we hypothesize that kidney function, kidney volume or kidney weight (as proxies for nephron mass) determine uEV excretion and that nephrectomy reduces uEV excretion. To address this, we analyzed uEV excretion in 24‐hour and spot urines from kidney donors before and after nephrectomy and in rats before and after nephrectomy. We show that kidney function, kidney volume, and kidney weight are related to uEV excretion rate, but that nephrectomy causes a lower than expected decrease in uEV excretion due to hypertrophy.

## 2 METHODS

### 2.1 Donor nephrectomy study

The protocol for the prospective study in 19 kidney donors was approved by the medical ethics committee of the Erasmus Medical Center (MEC‐2017‐068), and all participants provided informed consent. The only exclusion criterion was inability to comply with the study procedure. Participants were requested to collect 24‐h urine and spot urine before the donor nephrectomy. These urine collections were repeated during a follow‐up visit three months after the donor nephrectomy. The urine samples were processed immediately after collection. A protease inhibitor cocktail (Roche cOmplete protease inhibitor cocktail tablets, Switzerland) was added to spot urines, but not to the 24‐h urines because of the large number of tablets required for this volume. All urines were centrifuged at 2000*g*, for 10 min at 4˚C and then immediately aliquoted and stored at ‐80˚C until further analysis. Routine laboratory parameters were measured by the Department of Clinical Chemistry of the Erasmus MC. Estimated glomerular filtration rate (eGFR) was calculated using the CKD‐EPI equation and adjusted for body surface area.

### 2.2 Total kidney volume

Baseline CT scans were performed before donor nephrectomy as part of the standard clinical work‐up. CT images were analyzed using commercially available post‐processing software (Intellispace, Philips, the Netherlands). Using a standard multiplanar viewer, imaging planes, fixed at 90˚‐angles to each other, were aligned with the long and short axis of the kidney. Total kidney volume (TKV) was measured by segmenting the cortex and parenchyma using a smart segmentation tool in the “tumor tracking” application in the Intellispace software. Renal cysts, the pyelum and vascular structures were excluded from the segmentation. The total volume of the segmented kidney was automatically calculated by the software. Measurements were performed for both kidneys by a radiologist (R.B.).

### 2.3 Nephrostomy drain study

To compare uEVs derived from the kidney and the urological tract we also performed a study in patients with a unilateral nephrostomy drain (MEC‐2016‐069). Exclusion criteria were kidney replacement therapy, neobladder, and urogenital cancer. Timed urine samples were collected from the nephrostomy drain and from normal micturition (“bladder” samples). Urine samples were processed as described above and then prepared for quantification with EVQuant and uEV isolation with differential ultracentrifugation (see below). Finally, uEVs were analyzed pair‐wise by liquid chromatography and mass spectrometry (LC‐MS/MS) using a Q Exactive mass spectrometer (Thermo Fisher Scientific, Rockford, IL), as described previously (Salih, Demmers, et al., 2016). For quantitative analysis, individual peptide samples were TMT labelled (TMT10plex isobaric label reagent set, Thermo Fisher Scientific). The DAVID bioinformatics resource (version 6.7) was used to calculate Gene Ontology term enrichment.

### 2.4 Rat nephrectomy studies

Rat nephrectomy studies were performed to correlate kidney weight with uEV excretion. These studies were approved by the Animal Welfare Committee of the Erasmus Medical Center (16‐790‐02) (Bovée et al., 2021). Briefly, male Sprague Dawley rats (6 weeks old, average weight 200 g) were randomly assigned to undergo sham surgery (*n *= 10), uninephrectomy (*n *= 8), or 5/6^th^ nephrectomy (*n* = 8). 5/6^th^ nephrectomy was performed in two steps, including right uninephrectomy followed by surgical excision of the upper and lower poles of the left kidney 10 days later. Before and 8 weeks after nephrectomies GFR was measured by transcutaneous measurement of fluorescein isothiocyanate (FITC)‐sinistrin clearance, as reported previously (Schock‐Kusch et al., 2009). Both before and 8 weeks after surgery, 24‐h urine was collected using metabolic cages with protease inhibitor tablets in the urine collection reservoir. All urines were stored at ‐80˚C after centrifugation (2000*g*, 10 min at 4˚C). During the entire study the rats were on regular chow and had free access to water.

### 2.5 Nanoparticle tracking analysis

NTA was performed using a NanoSight NS300 (Sysmex, The Netherlands) with NTA 3.1 software (NanoSight, UK). Whole urine samples were vortexed and diluted in phosphate buffered saline (PBS, pH 7.4, 137 mM Na^+^, used throughout the studies) to obtain 40–100 particles per field, then inserted in an O‐ring top plate NTA chamber with a syringe. Particle scattering of 405 nm light was recorded by a CCD camera (five videos of 30 s each, camera level = 14, detection threshold = 3), and the Brownian motion was determined frame to frame. The lower limit of detection was approximately 70 nm.

### 2.6 EVQuant

EV quantification was performed using a newly developed assay (EVQuant) (Hartjes et al., 2020). Briefly, the whole urine sample was labelled with CD9‐Alexa647 (Table S1) in 0.03% w/v bovine serum albumin for 2 h, then diluted threefold in PBS and non‐specifically labelled by the generic fluorescent membrane dye Rhodamine R18 (0.33 ng/μl, 568 nm). Subsequently, without any isolation or purification procedures, the labelled samples were mixed with a non‐denaturing polyacrylamide gel solution (final ratio of 16% w/w acrylamide/bisacryl). The mixtures were transferred to a 96‐wells plate (SensoPlate glass bottom 96‐well plate, Austria). Immobilized uEVs were imaged using a spinning disk confocal microscope system (Opera Phenix, Perkin Elmer, USA). uEV concentration was corrected by dye in control solution (PBS). In this analysis, each detected EV (Rhodamine‐R18 +) was assessed for CD9 expression. The detection threshold (mean plus three times the standard deviation) was determined using a 100 nm liposome sample lacking protein markers.

### 2.7 CD9–TR‐FIA

The CD9–TR‐FIA was performed as previously described (Duijvesz et al., 2015). Briefly, a white neutravidin‐coated plate (Life Technologies) was coated with biotinylated anti‐human CD9 (1:500, EBioscience, USA) overnight at 4˚C (Table S1). 100 μl of thawed urine was vortexed and added to incubate for 1 h at room temperature. Next, Europium‐conjugated anti‐CD9 (0.25 ng/μl, CellGS, United Kingdom) was added and incubated for 1 h at room temperature. Incubation steps were performed on a plate shaker and were followed by six washes with wash buffer (Kaivogen, Finland). Before signal measurement on a Victor 1420 multilabel counter, a Europium enhancer (Kaivogen, Finland) was added to the empty well and incubated for 15 min in the dark.

### 2.8 Differential ultracentrifugation and immunoblotting

A “200K pellet” was obtained with ultracentrifugation using 50 ml whole urine as starting volume. Briefly, after a 17,000*g* spin (to remove remaining whole cells, large membrane fragments, and other debris), the pellet was dissolved in 250 μl freshly made 200 mg/ml dithiothreitol (DTT) diluted in ddH_2_O, heated and added to isolation buffer (10 mM triethanolamine, 250 mM sucrose, pH 7.6) and again centrifuged at 17,000*g*. The two supernatants were combined and centrifuged at 200,000*g* for 2 h. The 200K pellet was suspended in PBS, 6X Laemmli solution (440 mM Tris HCl pH 6.8, SDS 10% w/v, glycerol 25% v/v, bromphenolblue 0.1% w/v, β‐mercaptoethanol 6% v/v) was added and the sample was heated for 10 minutes at 60˚C. SDS‐PAGE was carried out on a gradient gel (4%–12% Criterion precast gel, 26 well, 15 μl, Bio‐Rad, USA) and transferred to PVDF membranes (0.2 μm PVDF, Bio‐Rad, USA) using a Trans‐Blot Turbo Transfer system (Bio‐Rad, USA) at 25V, 1A during 30 min. The membranes were blocked (TBS with 0.1% v/v Tween‐20 and 5% w/v BSA or milk) and probed overnight at 4°C with a primary antibody (Table S1). Subsequently, membranes were washed and incubated with a secondary antibody. After washing of the membranes, they were exposed to enhanced chemiluminescence substrate (Clarity Western ECL substrate, Bio‐Rad, USA) and analyzed by an Amersham system (GE Life Sciences, USA). uEV protein abundances were normalized by urine creatinine and analyzed with and without adjustment for TKV.

### 2.9 Statistical analysis

The data were analyzed for normal distribution and the statistical tests were selected accordingly. The data are expressed as means with standard deviations or medians with interquartile range (IQR), as appropriate. Comparisons between males and females were performed using Student's T‐test. Correlations were analyzed by Pearson or Spearman's rho (R). Comparisons before and after nephrectomy were performed using paired T‐test or Wilcoxon signed rank test. Intra‐individual comparisons were performed in spot urines using uEV/creatinine (Blijdorp et al., 2021). For inter‐individual comparisons with spot urines a “calculated spot uEV excretion” was determined by multiplying the uEV‐to‐creatinine ratio in spot urine by 24‐hour urinary creatinine: calculated spot urine uEV excretion (uEVs/min) = spot uEV concentration (uEVs/L) × creatinine excretion (mmol/min)/spot urine creatinine concentration (mmol/L). This approach has been used previously to estimate 24‐h sodium excretion from spot urine (Dong et al., 2019), and corrects for differences in creatinine excretion caused by variation in muscle mass. In addition, we validated this equation with data from a previous study (Blijdorp et al., 2021) with which we calculated spot uEV excretions (*n *= 8) and compared these to measured 12‐h uEV excretions; this analysis showed high correlations (*R* ^2^ 0.79–0.99). The uEV protein comparisons were performed by Mann‐Whitney test with correction for multiple testing using the Holm‐Bonferroni method. A *p*‐value ≤0.05 was considered statistically significant.

## 3 RESULTS

### 3.1 Female donors excrete fewer uEVs

**FIGURE 1:**
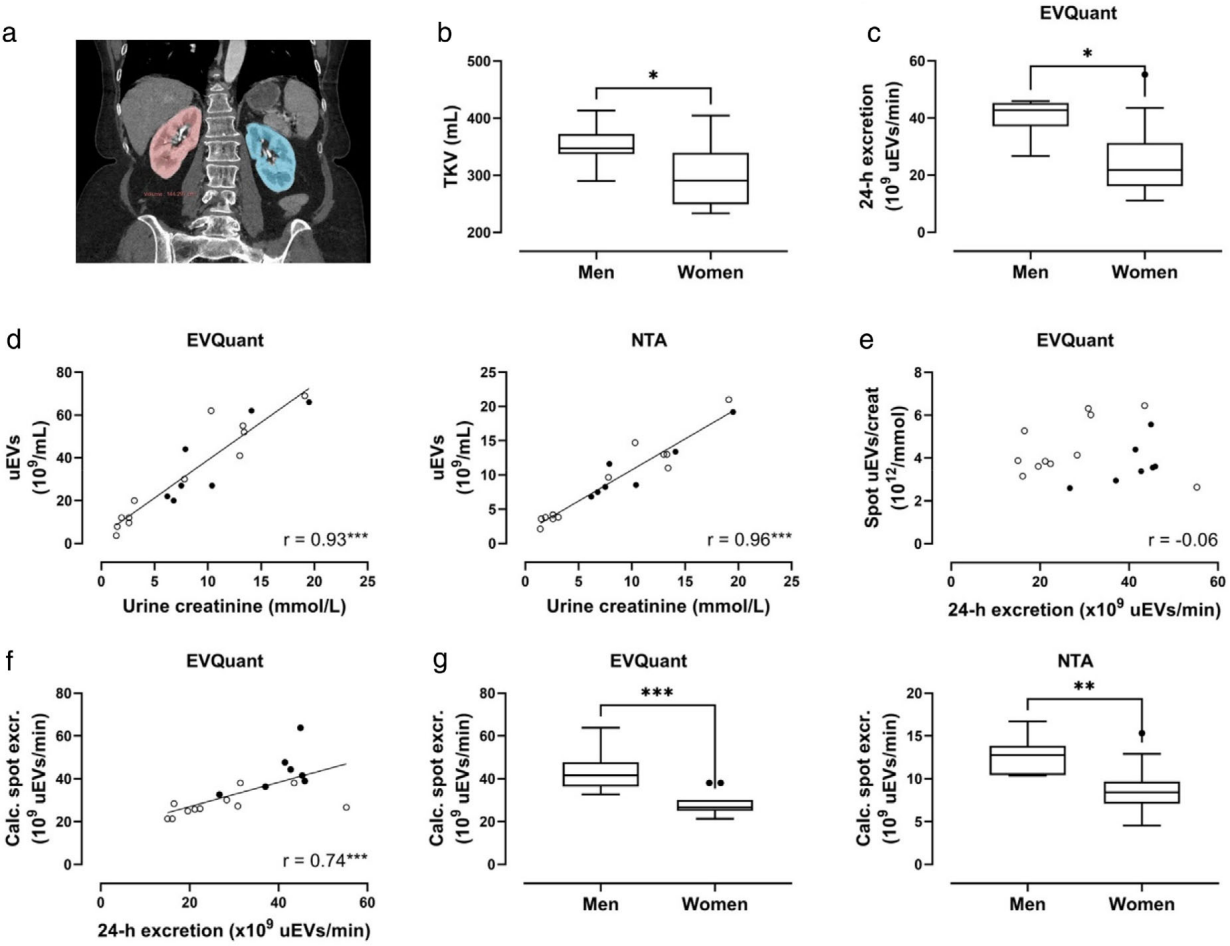
Total kidney volume and urinary extracellular vesicle (uEV) excretion in males and females. (a) Example of total kidney volume determination by segmentation in a computed tomography (CT) image of the kidneys (right kidney red, left kidney blue); (b) Comparison of total kidney volume (TKV) between men and women; (c) uEV excretion in men versus women measured by EVQuant in 24‐h urine; (d) Pearson correlations of urine creatinine versus uEV concentration in men (●) and women (○), measured by EVQuant (left panel) and nanoparticle tracking analysis (NTA, right panel); (e) Spearman correlation of 24‐h uEV excretion versus spot uEV/creatinine measured by EVQuant; (f) Spearman correlation of 24‐h uEV excretion versus calculated spot uEV excretion (spot uEV/creatinine * 24‐h urine creatinine) measured by EVQuant; (g) Calculated spot uEV excretion in men versus women measured by EVQuant (left panel) and NTA (right panel). Box plots are Tukey plots. Men are represented by ● and women by ○; *p ≤ 0.05, **p < 0.01, ***p < 0.001

**FIGURE 2:**
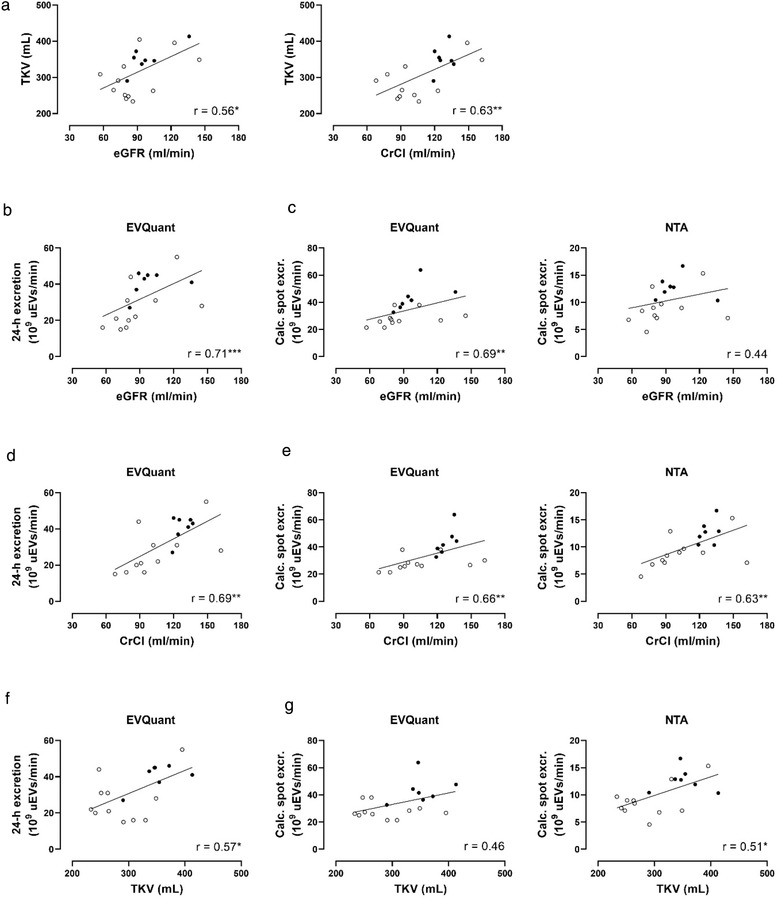
Correlations between total kidney volume, kidney function, and urinary extracellular vesicle (uEV) excretion. (a) Pearson correlations of total kidney volume (TKV) with estimated glomerular filtration rate (eGFR, corrected for body surface area), and creatinine clearance (CrCl); (b) Pearson correlation of 24‐h uEV excretion and eGFR; (c) Spearman correlations of calculated spot uEV excretions (measured by EVQuant or NTA) with eGFR; (d) Spearman correlation of 24‐h uEV excretion and CrCl; (E) Spearman correlations of calculated spot uEV excretions (measured by EVQuant or NTA) with CrCl; (f) Spearman correlation of 24‐h uEV excretion and TKV; (g) Spearman correlations of calculated spot uEV excretions (measured by EVQuant or NTA) with TKV. Men are represented by ● and women by ○; *p ≤ 0.05, **p < 0.01, ***p < 0.001

**FIGURE 3:**
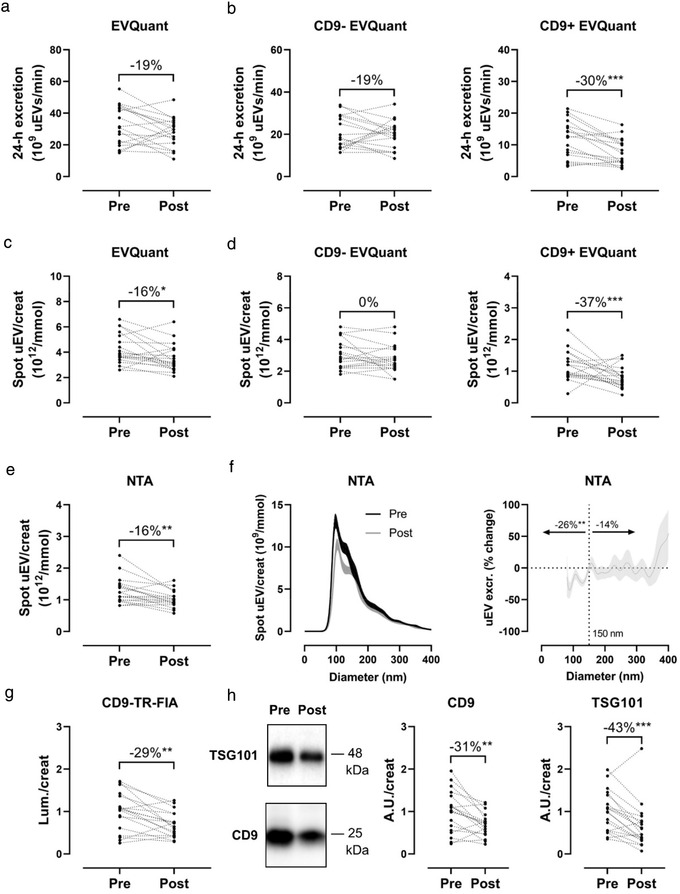
Effect of donor nephrectomy on urinary extracellular vesicle (uEV) excretion. (a) uEV excretion before (Pre) versus after (Post) donor nephrectomy measured by EVQuant in 24‐h urine; (b) Urinary excretion of CD9‐ uEVs (left panel) and CD9+ uEVs (right panel) before and after donor nephrectomy measured by EVQuant in 24‐h urine; (c) Spot uEV/creatinine before and after donor nephrectomy measured by EVQuant; (d) Spot uEV/creatinine ratios of CD9‐ (left panel) and CD9+ uEVs (right panel) before and after donor nephrectomy measured by EVQuant; (e) Spot uEV/creatinine before versus after donor nephrectomy measured by nanoparticle tracking analysis (NTA); (f) Size distribution of uEVs by NTA (left panel) and percentage change of size distribution (right panel, uEV/creatinine ratio ± SEM per 1 nm size bin; (g) CD9‐Europium signal to urine creatinine ratio (Lum./creat) before and after donor nephrectomy measured by CD9–TR‐FIA (signal pre‐donation normalized to 1); (h) Representative immunoblots of CD9 and TSG101 in the 200K uEV pellet before and after donor nephrectomy, loaded relative to individual urine creatinine concentrations, with corresponding densitometry. *p ≤ 0.05, **p < 0.01, ***p < 0.001

Nineteen subjects (12 females, seven males, Table 1) were analyzed before (Figures 1 and 2) and after (Figure 3) donor nephrectomy. One 24‐h urine collection was discarded because it was incomplete (as indicated by the participant and confirmed by a discrepancy between creatinine clearance and eGFR). Compared to male donors, female donors had a lower urinary creatinine excretion (9.0 ± 2.6 vs. 17.1 ± 1.3 mmol/day, *p* < 0.001), lower creatinine clearance (99 ± 33 vs. 128 ± 7 ml/min, *p* = 0.04), and lower TKV (300 ± 57 ml vs. 351 ± 37 ml, *p* = 0.05); eGFR was not significantly different (89 ± 25 vs. 99 ± 18 ml/min, *p* = 0.4; Figure 1a‐b, Table S2). Female donors excreted 49% fewer uEVs per day than male donors (22 [IQR 16–31] vs. 43 [IQR 37–45] × 10^9^ uEVs/min, *p* < 0.05, Figure 1c). In spot urines, creatinine concentrations strongly correlated with uEV concentrations measured with EVQuant or NTA (Figure 1d). Spot uEV/creatinine, however, did not correlate with 24‐h uEV excretion measured with EVQuant (Figure 1e). Calculated spot uEV excretion (multiplying spot uEV/creatinine by 24‐h urinary creatinine excretion) did correlate with measured 24‐h uEV excretion (Figure 1f). When calculated spot uEV excretions were compared between men and women (Figure 1g), the lower uEV excretion in women was similar to the analysis with 24‐h uEV excretion (Figure 1c). In a Bland‐Altman analysis, calculated spot uEV excretion largely agreed with 24‐h uEV excretion (Figure S1).

**TABLE 1:** General characteristics of the kidney donors

Variable	Pre‐nephrectomy (*n* = 19)	Post‐nephrectomy (*n* = 19)	*p*‐value
**General characteristics**			
Age, years	58 ± 12	–	
Female sex, *n* (%)	12 (63)	–	
Body mass index, kg/m^2^	26 ± 5	27 ± 6	**0.004**
Plasma sodium, mmol/L	142 ± 2	141 ± 3	0.1
Plasma potassium, mmol/L	4.0 ± 0.4	4.3 ± 0.4	**0.02**
eGFR, ml/min^a^	91 ± 20	58 ± 14	**<0.001**
Creatinine clearance, ml/min	110 ± 29	66 ± 17	**<0.001**
**24‐h urine**			
Volume, ml	1981 ± 754	2393 ± 830	**0.01**
Osmolality, mOsm/kg	474 ± 252	432 ± 213	0.4
Creatinine, mmol/day	11.8 ± 4.7	11.5 ± 4.6	0.3
Sodium, mmol/day	132 ± 51	155 ± 75	0.2
Potassium, mmol/day	80 ± 34	76 ± 20	0.5
Protein, mg/day	82 ± 18	97 ± 32	0.07
**Spot urine**			
Osmolality, mOsm/kg	554 ± 263	442 ± 225	0.07
Creatinine, mmol/L	8.5 ± 5.7	7.0 ± 5.4	0.30
Protein to creatinine ratio, g/mmol	9.2 ± 4.8	10.2 ± 4.4	0.20

^a^Estimated glomerular filtration rate (eGFR) adjusted for body surface area.

### 3.2 Kidney function and volume determine uEV excretion rate

eGFR (adjusted by body surface area) and creatinine clearance correlated positively with TKV (Figure 2a). eGFR also correlated positively with measured 24‐h uEV excretion (Figure 2b) and calculated spot uEV excretions (Figure 2c), although the correlation with uEVs quantified by NTA was not statistically significant. In addition, creatinine clearance correlated positively with measured 24‐h uEV excretion (Figure 2d) and calculated spot uEV excretions (Figure 2e). TKV correlated positively with measured 24‐h uEV excretion (Figure 2f) and calculated spot uEV excretions (Figure 2g), although the correlation with uEVs quantified by EVQuant was not statistically significant. Of note, the correlations between eGFR, creatinine clearance and uEV excretion were slightly stronger than for TKV and uEV excretion.

### 3.3 Effect of donor nephrectomy on uEV excretion

Donor nephrectomy reduced eGFR from 91 ± 20 to 58 ± 14 ml/min (‐36% ± 10%) and creatinine clearance from 110 ± 29 to 66 ± 17 ml/min (‐38% ± 11%, *p* < 0.001 for both, Table 1). Plasma potassium significantly increased from 4.0 ± 0.4 to 4.3 ± 0.4 mmol/L (*p* = 0.02). The degree of proteinuria or albuminuria increased non‐significantly after donor nephrectomy. Body mass index did increase significantly but this was not caused by an increase in muscle mass (non‐significant decrease in 24‐h creatinine excretion). The donor kidney was most often the left kidney (13 out of 19) and had a similar TKV as the remaining kidney (158 ± 30 vs. 159 ± 30 ml), implying that nephron number was reduced by 50% ± 3%. Donor nephrectomy reduced uEV excretion (measured by EVQuant) by 19% (IQR ‐11 to 34), although this was not statistically significant (*p *= 0.09, Figure 3a). Interestingly, when classified by surface marker CD9, CD9‐ uEVs did not decrease significantly, whereas CD9+ uEVs decreased by 30% (IQR 20–48, *p* < 0.001, Figure 3b). This was also observed with spot uEV/creatinine which showed a significant decrease of 16% (IQR 4–26, *p *= 0.02) for all uEVs (Figure 3c) and 37% (IQR 17–58, *p* = 0.01) for CD9+ uEVs (Figure 3d). The same decrease was found when using NTA for uEV counts (16%, IQR 2–36, *p* = 0.003, Figure 3e), especially for smaller uEVs (<150 nm, 26%, IQR 10–45, *p* = 0.002, Figure 3f). The effect on CD9 was confirmed when using CD9–TR‐FIA for uEV quantification, which showed a decrease of 29% (IQR 13–49, *p* = 0.004, Figure 3g). Immunoblot analysis of the urinary exosome markers CD9 and TSG101 in uEVs showed a decrease of 31% (IQR 16–42, *p *= 0.004) and 43% (IQR 28–60, *p *= 0.003), respectively (Figures 3h and S2).

### 3.4 Donor nephrectomy causes nephron segment‐specific changes

**FIGURE 4:**
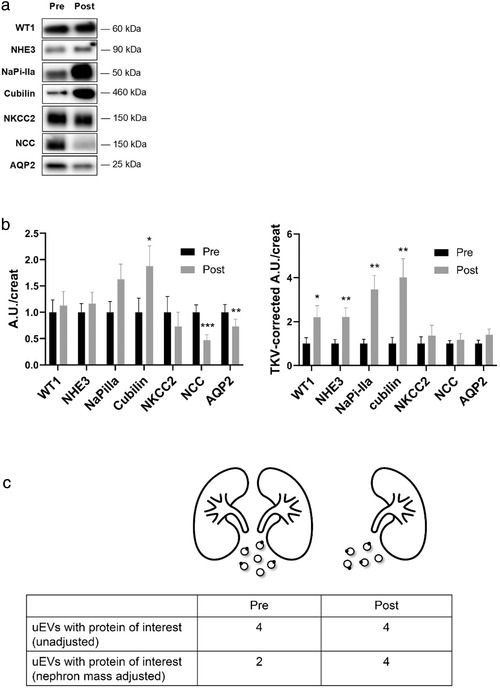
Changes in urinary extracellular vesicle (uEV) nephron marker proteins after donor nephrectomy. (a) Representative immunoblots before and after donor nephrectomy of nephron marker proteins in uEVs isolated from spot urine, including the podocyte marker Wilm's tumour 1 (WT1), the proximal tubule markers sodium‐hydrogen exchanger 3 (NHE3), sodium/phosphate co‐transporter IIa (NaPi‐IIa), and cubilin, the loop of Henle marker sodium‐potassium‐chloride co‐transporter 2 (NKCC2), the distal convoluted tubule marker sodium‐chloride cotransporter (NCC), and the collecting duct marker aquaporin‐2 (AQP2). All proteins were loaded relative to urine creatinine; (b) Densitometry of absolute changes in uEV protein abundances before (Pre) and after (Post) donor nephrectomy (*n *= 19; A.U., arbitrary units). Densitometry of uEV protein abundances relative to total kidney volume (TKV‐corrected A.U./creat) before (Pre) and after (Post) donor nephrectomy; c) Cartoon illustrating the changes in uEV and uEV biomarker excretion before (Pre) and after (Post) donor nephrectomy. Donor nephrectomy reduces the overall uEV excretion rate by ∼19% (6 to 5 uEVs in the cartoon). In the example, four out of six uEVs contain an uEV biomarker of interest before donor nephrectomy and four out of five uEVs after donor nephrectomy. This illustrates the need to normalize by kidney volume otherwise the doubling in uEV biomarker would remain undetected. This example illustrates the observations for WT1 and NHE3 (no change when not adjusting for total kidney volume versus two‐fold increase when adjusting for total kidney volume, Figure 4B). *p ≤ 0.05, **p < 0.01, ***p < 0.001

In addition to quantifying uEVs, we also analyzed nephron segment‐specific proteins in uEVs isolated from spot urines (Figures 4a and S2), including Wilm's Tumor 1 (WT1, a podocyte marker), sodium‐hydrogen exchanger 3 (NHE3), sodium/phosphate co‐transporter IIa (NaPi‐IIa), and cubilin (three proximal tubule markers), sodium‐potassium‐chloride co‐transporter 2 (NKCC2, loop of Henle marker), the sodium‐chloride cotransporter (NCC, distal convoluted tubule marker), and aquaporin‐2 (AQP2, collecting duct marker). Donor nephrectomy affected these nephron segment‐specific proteins in uEVs differently with a significant increase in cubilin and decrease in NCC and AQP2 abundance in uEVs (Figure 4b). Corrected for initial kidney volume, however, WT1 and the proximal tubule markers NHE3, NaPi‐IIa, and cubilin were significantly increased by a factor 2–4, while distal nephron markers did not change (Figure 4b). This implies that a two‐fold increase in an uEV‐related protein (e.g., NHE3) would remain undetected when not correcting for the nephrectomy (Figure 4c).

### 3.5 Comparison of uEVs derived from kidney versus bladder urine

**FIGURE 5:**
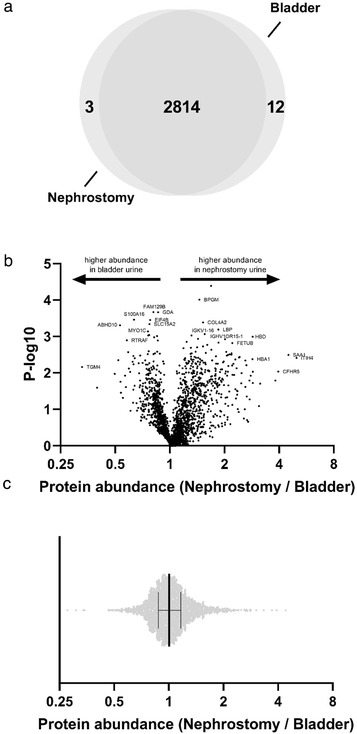
Comparison of the number and abundance of uEV proteins from nephrostomy or bladder urine. (a) In nine patients with a nephrostomy drain urine samples for uEV analysis were collected from both the nephrostomy drain and normal micturition (“bladder,” see also **Table** **S3** and **S4**). Mass spectrometry identified 2829 proteins of which 2814 were identified in uEVs isolated from the nephrostomy drain and bladder urines and 3 and 12 proteins were only identified in the nephrostomy and bladder urine samples, respectively. (b) A Vulcano plot is shown for the 2462 proteins for which the abundance could be determined indicating whether protein abundance was higher in the bladder or nephrostomy urine sample. (c) The distribution of the nephrostomy/bladder uEV protein abundance ratios is shown illustrating that the abundance of the majority of the uEV proteins was similar in both sources (nephrostomy/bladder uEV protein abundance ratio 1.0, IQR 0.9–1.2)

To compare the contribution of the kidney and the other parts of the urinary tract to overall uEV excretion, we performed a study in patients with a nephrostomy drain (see Tables S3 and S4 for patient and urine characteristics). This allowed a comparison between uEVs derived directly from the kidney (nephrostomy drain) with uEVs derived from the kidneys and urinary tract (“bladder”). The nephrostomy drain samples contained more uEVs than the bladder samples, although this was not statistically significant (7.4 ± 1.6 vs. 3.3 ± 1.0 × 10^10^ uEVs/min, *p* = 0.08). Mass spectrometry identified 2814 proteins that were present in both the nephrostomy and bladder samples, while three and 12 proteins were only identified in the nephrostomy and bladder samples, respectively (Figure 5a). Of all proteins identified, 66% was associated with extracellular exosomes. uEV proteins in the nephrostomy samples were enriched for extracellular region, blood microparticles, and immune response; uEV proteins in the bladder samples were enriched for membrane, extracellular exosome, and endocytosis proteins (*p* for all <0.001). The abundance of 2462 proteins could be determined by mass spectrometry (Table S5). When the abundance of these uEV proteins in the nephrostomy and bladder samples was compared, the abundance of the majority of the uEV proteins was similar in both sources (nephrostomy/bladder uEV protein abundance ratio 1.0, IQR 0.9–1.2, Figure 5b‐c).

### 3.6 Nephrectomy in rats reduces uEV excretion

**FIGURE 6:**
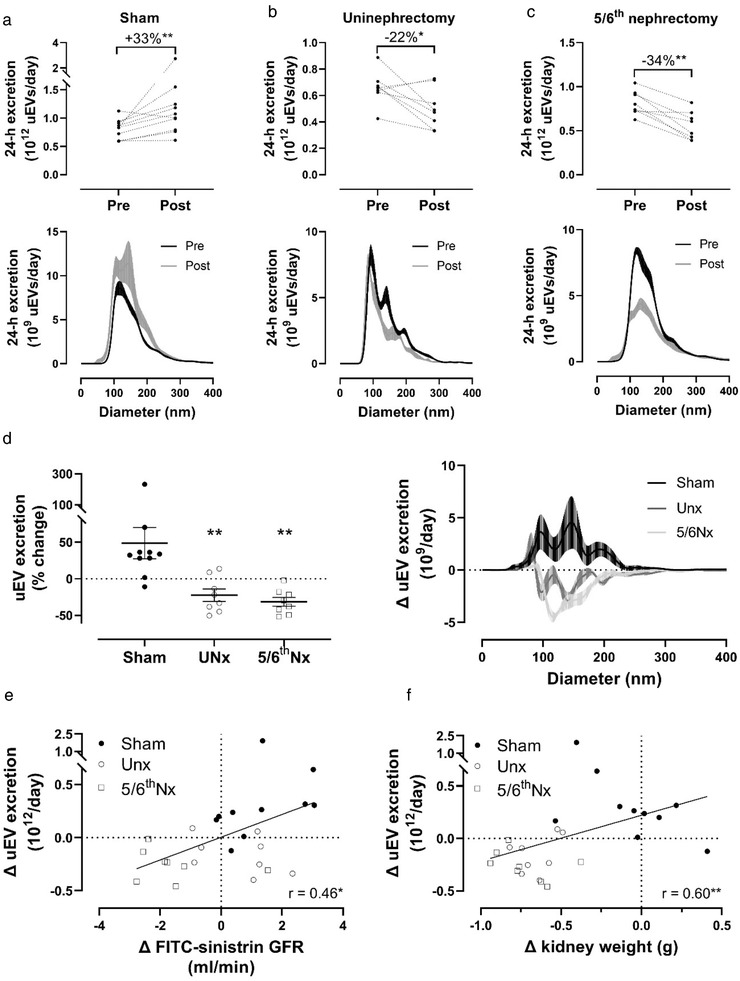
Effect of sham, uninephrectomy or 5/6^th^ nephrectomy on urinary extracellular vesicle (uEV) excretion in rats. (a) 24‐h uEV excretion before (Pre) and 8 weeks after (Post) sham surgery (*n =* 10) and uEV size distribution (right panel, ± SEM per 1 nm bin size); (b) 24‐h uEV excretion before and 8 weeks after uninephrectomy (*n =* 8) and uEV size distribution (lower panel, ± SEM per 1 nm bin size); (c) 24‐h uEV excretion before and 8 weeks after 5/6^th^ nephrectomy (*n =* 8) and uEV size distribution (lower panel, ± SEM per 1 nm bin size); (d) Percentage change in uEV excretion before‐after sham surgery, uninephrectomy or 5/6^th^ nephrectomy, including uEV size distribution (right panel, ± SEM per 1 nm size bin); (e) Spearman correlation between the change in uEV excretion and FITC‐sinistrin glomerular filtration rate (GFR); •, sham surgery; ○ uninephrectomy; □ 5/6^th^ nephrectomy; f) Spearman correlation between the change in 24‐h uEV excretion and kidney weight. *p ≤ 0.05, **p < 0.01, ***P < 0.001

uEV excretions were also analyzed by NTA in rats before and eight weeks after sham surgery, uninephrectomy (as comparison for donor nephrectomy) or 5/6^th^ nephrectomy (a commonly used model for chronic kidney disease) (Bovée et al., 2021). Baseline uEV excretion was similar between the three groups (8.3 [IQR 6.3–9.0], 6.6 [IQR 6.4–6.8] and 7.7 [IQR 7.3–9.1] × 10^11^ uEVs/day, *p* = 0.09). Eight weeks after sham surgery, an increase in body weight (183 ± 22 g), FITC‐GFR (2.4 ± 0.5 to 3.7 ± 1.0 ml/min, 65% ± 63%), and uEV excretion (33%, IQR 28%–36%) was observed; the sham operation did not affect uEV size distribution (Figure 6a).

Eight weeks after uninephrectomy body weight increased similarly as in sham rats (186 ± 21 g), while the increase FITC‐GFR was less (from 3.8 ± 0.7 to 4.4 ± 1.1 ml/min, 19% ± 33%), and uEV excretion decreased by 24% ± 6% (*p* = 0.04, Figure 6b). Eight weeks after 5/6^th^ nephrectomy body weight increased by 145 ± 20 g (less than sham, *p *= 0.002), whereas FITC‐GFR decreased from 3.3 ± 0.6 to 1.3 ± 0.4 ml/min (‐61% ± 13%, 76% lower than sham), and uEV excretion decreased by 34% ± 6% (Figure 6c). This decrease especially concerned uEVs with a diameter between 100 and 200 nm (Figure 6c). In a direct comparison, uEV excretion was significantly lower in rats after uninephrectomy and 5/6^th^ nephrectomy compared with sham‐operated rats (*p *< 0.001 for both), especially regarding uEVs between 100 and 200 nm (Figure 6d). The change in FITC‐sinistrin GFR correlated with the change in uEV excretion (*R* = 0.46, *p* < 0.01, Figure 6e). Furthermore, the change in kidney weight correlated with the change in uEV excretion (*R* = 0.60, *p* < 0.01, Figure 6f). Finally, the estimated degree of hypertrophy matched the change in uEV excretion rate in the remaining kidney tissue (Table 2).

**TABLE 2:** Estimated hypertrophy and change in uEV excretion after rat nephrectomy

		Uninephrectomy	5/6^th^ nephrectomy
**Kidney**	Kidney weight at uninephrectomy, grams	1.2 ± 0.0	1.2 ± 0.1
	Terminal kidney weight, grams	1.7 ± 0.1	1.6 ± 0.2
	Projected weight remaining kidney without hypertrophy, grams	1.2	1/3 × 1.2 = 0.4
	Estimated degree of hypertrophy^a^	1.4‐fold	4‐fold
**uEVs**	uEV excretion at baseline, 10^12^ uEVs/day	0.66 ± 0.13	0.81 ± 0.14
	uEV excretion at sacrifice, 10^12^ uEVs/day	0.50 ± 0.15	0.56 ± 0.16
	Projected uEV excretion without hypertrophy^b^	1/2 × 0.66 = 0.33	1/6 × 0.81 = 0.14
	Estimated change in uEV excretion rate in remaining kidney tissue^c^	1.5‐fold	4‐fold

^a^ Calculated by (terminal kidney weight) / (projected weight remaining kidney).

^b^ Assuming both kidneys contribute equally and with limited extra‐renal contribution to uEVs.

^c^ Calculated by (uEV excretion at sacrifice) / (projected uEV excretion without hypertrophy).

## 4 DISCUSSION

Here, we show that uEV excretion is related to eGFR, creatinine clearance, TKV, and kidney weight, and that nephrectomy reduces uEV excretion less than would be expected based on the loss of nephron mass. Our findings have two important implications for the evolving field of uEV biomarker research and its clinical applicability, namely that (1) nephron mass or uEV excretion should be included for inter‐individual comparisons and (2) changes in uEV excretion rate are disproportional to nephron loss after nephrectomy due to compensatory hypertrophy.

We identified a sex difference in uEV excretion with women excreting 49% fewer uEVs. This is likely explained by higher nephron endowment in men. Although prostate‐derived uEVs could also contribute to this difference, these are usually low abundant except after digital rectal examination (Duijvesz et al., 2015; Ploussard & De La Taille, 2010). The identification of this sex difference in uEV excretion implies that when the excretion rate of a uEV biomarker is studied in males and females, the results should be corrected for uEV excretion to avoid under‐ or overestimation of biomarker levels.

The sex difference in urinary creatinine excretion likely explains why a previous study concluded that females excrete *more* EVs than males based on spot uEV/creatinine assessment alone (Turco et al., 2016). We do show that uEV excretion can be estimated using spot urines when multiplying spot uEV/creatinine by 24‐h urinary creatinine, an approach that has been used previously for predicting urinary sodium excretion (Dong et al., 2019). In addition to sex, genes involved in nephron endowment and age‐related glomerulosclerosis cause considerable variation in nephron number between healthy individuals (Bertram et al., 2011; Puelles et al., 2011), and therefore likely also in uEV excretion rate. The dependence on nephron mass has been shown previously for other urinary biomarkers such as uromodulin (Pivin et al., 2018). This implies that for inter‐individual comparisons of uEV‐biomarkers, an adjustment for nephron mass or uEV excretion is required. This may also explain why a previous study reported a lack of correlations between markers in kidney tissue and uEVs (Blijdorp & Hoorn, 2019; Sabaratnam et al., 2019), although other studies did identify such correlations (Bazzell et al., 2018; Wu et al., 2021). Of note, kidney‐uEV correlations may also vary because proteins are processed differently into uEVs. The strong inter‐individual correlation between urine creatinine and uEV concentration may be explained by the association between creatinine excretion (muscle mass) and measured GFR (nephron mass) (Tynkevich et al., 2015).

After human kidney donor nephrectomy or rat nephrectomy, the reduction in uEV excretion was lower than expected based on the reduction in kidney mass. This implies that the compensatory hypertrophy that is known to occur after nephrectomy (Brenner et al., 1972; Hayslett et al., 1968; Wessely et al., 2014) contributes to the relative increase in uEV excretion . Indeed, in our rat studies, the estimated degree of hypertrophy matched the fold‐change in uEV excretion rate suggesting that hypertrophied tissue is capable of secreting uEVs. These findings also have implications for uEV biomarker research. This was illustrated by our analysis of nephron‐specific markers in uEVs before and after donor nephrectomy. Without correction for the loss of nephron mass after the donor nephrectomy, the nephron‐specific marker profile in uEVs was completely different than with this correction. Of further interest was that donor nephrectomy especially affects the CD9+ population of uEVs. Previously we showed that the uEV abundance of CD9 was significantly reduced in patients with CKD stages G2‐4 compared with healthy controls (Salih, Demmers, et al., 2016). According to the Kidney Tubules Expression Atlas, CD9 is increasingly expressed in the distal nephron, but is virtually absent in the proximal tubule (Limbutara et al., 2020). Indeed, we recently showed that CD9 does not immunoprecipitate with NHE3 and NaPi‐IIa in uEVs (Blijdorp et al., 2021). Together, this suggests that the proximal tubule undergoes more hypertrophy than downstream nephron segments after uninephrectomy causing more CD9‐ uEV excretion (100% increase after correction for nephrectomy). This impression was confirmed by showing that several proximal tubule markers were upregulated in uEVs when correcting for nephron loss. This is also in agreement with previous work showing that uninephrectomy increases NHE3 activity and abundance by compensatory cell growth in mice (Girardi et al., 2002; Nord et al., 1985; Pollock et al., 1992). This is likely caused by chronic hyperfiltration (Preisig & Alpern, 1991), and is clinically relevant because it was previously linked to the development of salt‐sensitive hypertension in sheep after fetal uninephrectomy (Lankadeva et al., 2012; Singh et al., 2010), and in rats after adult uninephrectomy (Jung et al., 2011). While some epidemiological studies show that kidney donors remain normotensive after donation (Kasiske et al., 1995), others find an increase in blood pressure (Boudville et al., 2006; Lenihan et al., 2015), which could be a consequence of increased proximal tubular salt reabsorption (Gurley et al., 2011; Veiras et al., 2017). Increased proximal tubular salt reabsorption would be expected to reduce distal sodium delivery, which could impair distal potassium secretion. This may explain the minor rise in plasma potassium after kidney donation, an observation that has been made previously (Kasiske et al., 2015).

We also performed a study in patients with a nephrostomy drain in order to compare uEVs from urine directly derived from the kidney to urine derived from the complete urinary tract. In this analysis it was striking to see that the vast majority of uEV proteins was identified in both types of urine. Three possible explanations for this observation are that (1) the kidney is the main source of uEVs; (2) the kidney and the other parts of the urinary tract secrete the same proteins in uEVs; (3) plasma‐derived EVs contribute to uEVs. Although the protein identification analysis cannot differentiate between these options, our analysis of uEV protein abundances did show that the quantitative contribution of the post‐kidney urinary tract is limited. Although systemically infused EVs can reach the urine (Oosthuyzen et al., 2016), the quantitative contribution of plasma‐derived EVs also appears to be limited as a recent analysis showed that only 2 of 5113 uEV proteins were not detected at the RNA level in the urinary tract (Svenningsen et al., 2020).

This study is only the first step in establishing the determinants of uEV excretion, and therefore a number of limitations should be acknowledged. First, we did not measure GFR in the kidney donors as this is not routinely performed in our center. Ideally, uEV excretion would be compared to measured GFR and a true estimation of nephron number, for example by combining unenhanced computed tomography and biopsy‐based stereology (Sasaki et al., 2019). Similarly, linking a spot urine to the time of last void could facilitate the use of a timed uEV excretion for spot urines. Second, for this initial study, we chose a relatively “clean” model of nephron loss, that is, surgical removal of nephrons either by donor nephrectomy or 5/6^th^ nephrectomy. Future studies should address how uEV excretion changes over time during other forms of nephron loss, for example, progression of CKD. Finally, we acknowledge that not all particles are uEVs (Welsh et al., 2020), but this limitation mainly pertains to NTA and not EVQuant and CD9–TR‐FIA (Blijdorp et al., 2021).

Taken together, our data show that uEV excretion depends on nephron mass, and that nephrectomy reduces uEV excretion less than expected based on nephron loss due to compensatory hypertrophy. The major implication is that a measure for nephron mass or uEV excretion rate should be included when comparing uEV biomarkers between individuals.

## DISCLOSURE STATEMENT

The authors have nothing to disclose.

## Supporting information

Supporting Information

Supporting Information
