# Supplementary material for: Nephron mass determines the excretion rate of urinary extracellular vesicles
Source: J Extracell Vesicles. 2022 Jan 22;11(1):e12181. doi: 10.1002/jev2.12181 (PMC8783354; doi:10.1002/jev2.12181)

Supplemental Material

Nephron mass determines the excretion rate of urinary extracellular vesicles

Charles J. Blijdorp MD, Thomas A. Hartjes MSc, Dominique M. Bovée MD,

Ricardo P.J. Budde MD, PhD, Jacqueline van de Wetering MD, PhD,

Joost G.J. Hoenderop PhD, Martin E. van Royen PhD, Robert Zietse MD, PhD,

David Severs MD, Ewout J. Hoorn MD, PhD

Table of content:

Table S1 - Antibodies

Table S2 - Comparison of baseline characteristics in female and male kidney donors

Table S3 - Patient characteristics nephrostomy drain study

Table S4 - Characteristics urine samples from nephrostomy drain and bladder

Figure S1 - Bland-Altman calculated spot vs. 24-hour uEV excretion

Figure S2 - Full immunoblots

Table S1. Antibodies

Techniques	Antibody	Type	Species	Concentration	Source	Cat#/clone
**EVQuant**	CD9 alexa-647	Primary	Mouse	1:25	Thermo SC	MA5-18154
**CD9–TR-FIA**	CD9-biotin	Capture	Mouse	1:500	Bioscience	SN4 C3-3A2
	CD9-Europium	Primary	Mouse	0.25ng/µL	CellGS	CGS12A
**Immunoblot**	WT1	Primary	Mouse	1:1000	Ventana	6F-H2
	CD9	Primary	Mouse	1:500	Bioscience	MAB1880
	TSG101	Primary	Mouse	1:333	Abcam	ab83
	Cubilin	Primary	Rabbit	1:12,000	Dr. Christensen	-
	NHE3	Primary	Rabbit	1:1000	Stressmarq	7644
	NaPi-IIa	Primary	Rabbit	1:500	Abcam	Ab83
	NKCC2	Primary	Rabbit	1:1000	Stressmarq	Spc-401D
	NCC	Primary	Rabbit	1:2000	Millipore	3553
	AQP2	Primary	Rabbit	1:1000	Stressmarq	9398
	Mouse HRP	Secondary	Goat	1:3000	Biorad	L005680
	Rabbit HRP	Secondary	Goat	1:3000	Biorad	L005679

Table S2. Comparison of baseline characteristics in female and male kidney donors

**Variable**	**Females (n = 12)**	**Males (n = 7)**	***P*-value**
Age, years	61 ± 11	52 ± 12	0.1
Body mass index, kg/m^2	26 ± 7	25 ± 2	0.7
eGFR, ml/min*	89 ± 25	99 ± 18	0.4
Creatinine clearance, ml/min	99 ± 33	128 ± 7	0.04
Plasma sodium, mmol/L	143 ± 2	142 ± 2	0.4
Plasma potassium, mmol/L	4.0 ± 0.3	4.3 ± 0.3	**0.05**
**24h urine**			
Volume, mL	2096 ± 671	1581 ± 803	0.2
Osmolality, mOsm/kg	334 ± 130	734 ± 216	**< 0.001**
Creatinine, mmol/day	9.0 ± 2.6	17.1 ± 1.3	**< 0.001**
Sodium, mmol/day	109 ± 33	173 ± 44	**0.002**
Potassium, mmol/day	63 ± 23	101± 39	**0.01**
Protein, mg/day	80 ± 21	83 ± 18	0.8
**Spot urine**			
Protein to creatinine ratio, g/mmol	10.5 ± 5.5	7.1 ± 2.6	0.1

* Estimated glomerular filtration rate (eGFR) adjusted for body surface area.

Table S3. Patient characteristics nephrostomy drain study

#	Sex, M/F	Age, years	eGFR, ml/min/1.73m^2	Reason for nephrostomy drain	Time drain placed, days	Collection time, min.
1	M	54	67	Kidney stones	1	105
2	F	30	118	Abscess	78	105
3	M	62	58	Colorectal cancer	27	135
4	F	62	35	Endograft	14	90
5	F	80	83	Colorectal cancer	55	120
6	M	86	22	Colorectal cancer	47	180
7	F	60	47	Metastatic cancer	38	N/A
8	M	59	99	Colorectal cancer	33	105
9	F	36	53	Kidney stones	27	90

Table S4. Characteristics urine samples from nephrostomy drain and bladder

**Variable**	**Nephrostomy (*n* = 9)**	**Bladder (*n* = 9)**	***P*-value**
Collection time, min*	116 ± 30	116 ± 30	-
Creatinine clearance, ml/min*	31 ± 21	33 ± 18	0.60
Protein/creatinine, g/mmol	95 ± 56	68 ± 71	0.4
Albumin/creatinine, mg/mmol	69 ± 40	45 ± 52	0.4
Sodium excretion, µmol/min*	23 ± 10	23 ± 14	1.0
Potassium excretion, µmol/min*	11 ± 4	13 ± 7	0.4
Diuresis, ml/min*	0.3 ± 0.2	0.3 ± 0.1	0.5

* These data were available for 8 patients because collection time was not available in particpant #7.

Figure S1: Bland-Altman

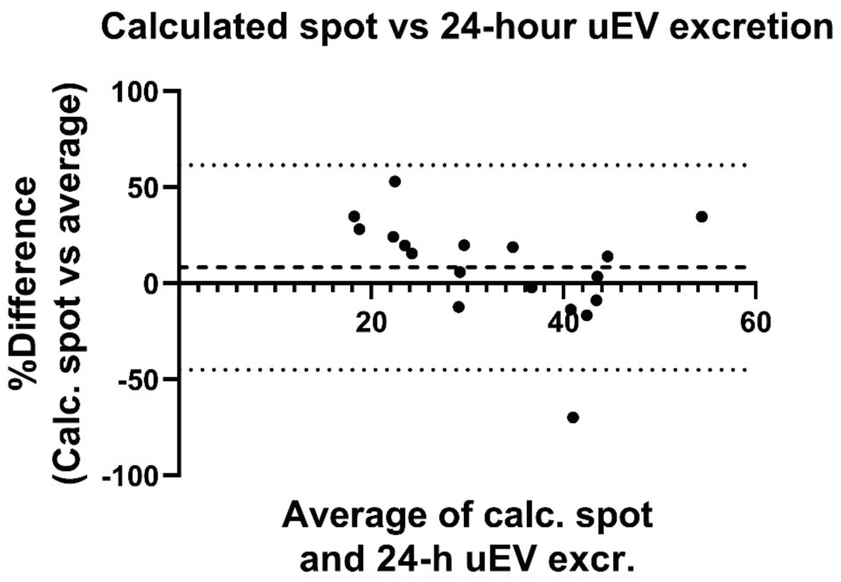

Legend Figure S1: Bland Altman analysis of calculated spot vs. 24-hour uEV excretion. The X-axis depicts the average of the two uEV excretion methods; on the Y-axis the percent difference of calculated spot excretion vs. the average of the uEV excretion methods is shown. Shown are Bias (---) and 95% Limits of agreement (….).

Figure S2: Full immunoblots

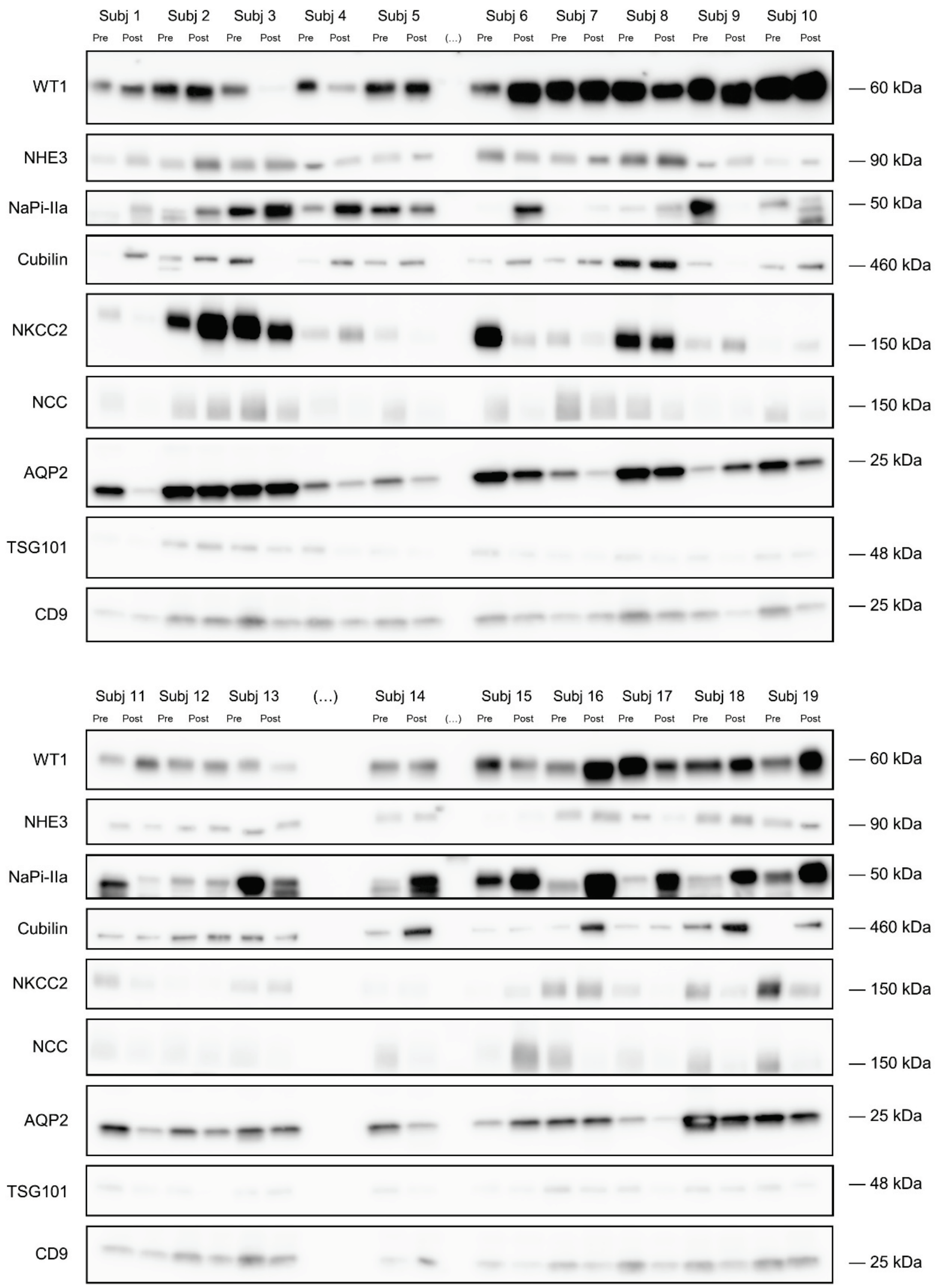

Supplement: Supplementary file 1 — Supporting Information [file JEV2-11-e12181-s002.pdf]
